# Long-term efficiency of pulmonary rehabilitation in patients with chronic obstructive pulmonary disease, bronchiectasis, and asthma: Does it differ?

**DOI:** 10.55730/1300-0144.5644

**Published:** 2023-03-07

**Authors:** Mustafa Engin ŞAHİN, Seher SATAR, Pınar ERGÜN

**Affiliations:** Department of Chest Disease, Ankara Atatürk Sanatoryum Training and Research Hospital, Health Sciences University, Ankara, Turkey

**Keywords:** Asthma, bronchiectasis, COPD, long-term, obstructive lung disease, pulmonary rehabilitation

## Abstract

**Background and aim:**

The long-term effects of pulmonary rehabilitation (PR) and maintenance programs in obstructive pulmonary diseases have not been sufficiently investigated, particularly in diseases other than COPD. This retrospective study aimed to examine the long-term results of individualized comprehensive outpatient pulmonary rehabilitation in patients with obstructive pulmonary disease.

**Materials and methods:**

This study is a single-center, retrospective cohort study. Between 2010 and 2019, 269 patients with chronic airway obstruction were treated in our multidisciplinary PR center at a tertiary training and research hospital, and they were divided into three groups based on their diagnosis: COPD, bronchiectasis, and asthma. Patients’ perceptions of dyspnea, exercise capacity, inspiratory and peripheral muscle strength, body composition, quality of life, and psychosocial status were compared at the beginning, end, and 12th and 24th months of PR.

**Results:**

Improvements in dyspnea perception remained longer in asthmatics than in the other two groups. The increases in exercise capacity in the bronchiectasis and asthma groups lasted two years. All groups maintained their respiratory muscle strength gains at the end of the second year. Improvements in hand grip strength in the COPD and bronchiectasis groups have been sustained for two years, but in the asthma group, enhancements were lost in the second year. Even after the second year, quality of life was still better than the baseline in all groups, despite a worsening in the first year. However, groups anxiety and depression improvements were not sustained after the first year.

**Conclusion:**

The long-term effectiveness of PR in patients with bronchiectasis and asthma was similar to that of COPD patients. Therefore, multidisciplinary, comprehensive PR programs should be integrated into the management of patients with bronchiectasis and asthma. We also recommend structured follow-up programs to maintain gains and to detect the need for rerehabilitation.

## 1. Introduction

Pulmonary rehabilitation is a highly effective treatment for chronic respiratory diseases that leads to reduced exercise capacity, poor quality of life, and muscle weakness [1]. While a very high level of evidence supports the efficacy of PR in COPD, its effect on other obstructive pulmonary diseases has yet to be adequately defined. If patients who have completed PR do not continue to exercise regularly, their PR gains will decrease. Some benefits can be kept longer as time passes, while others are lost in earlier eras. It has been reported that some gains after PR with maintenance programs can continue for 12–24 months in patients with COPD [2–4]. Studies investigating the long-term effects of PR in other obstructive pulmonary diseases are very limited [5,6]. The longevity of PR gains depends on the content of the initial PR program, how maintenance therapy is structured, patient compliance, and practices that encourage patient compliance. Maintenance treatments mainly consist of exercise programs. Individually tailored maintenance exercise programs might be considered.

This study examined how much of the PR gains could be preserved after 24 months in COPD, bronchiectasis, and asthma patients who completed an individualized multidisciplinary comprehensive outpatient PR program and continued structured follow-up programs.

## 2. Materials and methods

**Figure 1:**
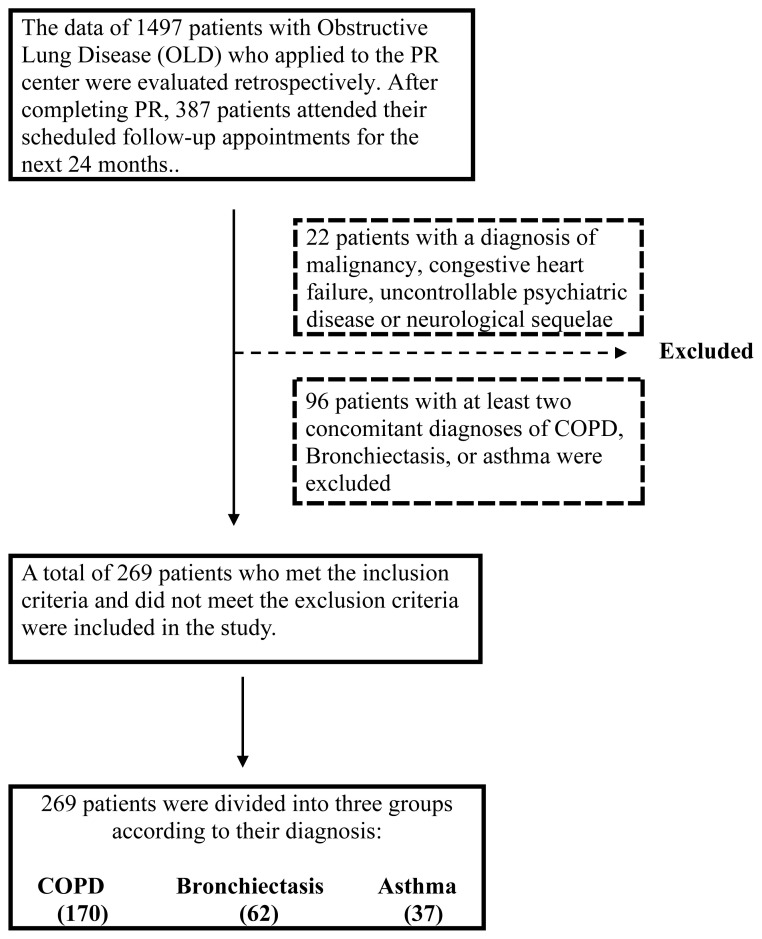
Patient selction.

This study is a single-center, retrospective cohort study. Written informed consent was obtained from all patients before the pulmonary rehabilitation program. Study approval was obtained from Ankara Atatürk Sanatoryum Training and Research Hospital, Medical Specialization Education Board Decision with document number 17-3, dated 16.12.2021. Patient selection is summarized in Figure 1.

**Inclusion criteria:**

- Having a diagnosis of COPD, bronchiectasis, or asthma
- Attending regular follow-up appointments for 24 months
- Declarination of continuing the maintenance program when one comes to the controls (The patients were asked if they continued their maintenance program during their controls, and this was recorded).

**Exclusion criteria:**

- Coexistence of at least two of the diagnoses of COPD, bronchiectasis, or asthma
- Diagnosis of malignancy, congestive heart failure, uncontrollable psychiatric disease, or neurological sequelae

As a result, after completing the multidisciplinary comprehensive outpatient PR program for 269 patients separated into groups based on COPD, bronchiectasis, and asthma diagnoses, the patients’ data were retrospectively examined to determine how much PR gains could be preserved in 24 months with a structured follow-up program. It is hypothesized that PR efficiency would be maintained in all three patient groups after 24 months.

### 2.1 Pulmonary rehabilitation

#### 2.1.1 Intensive program

PR was performed by a multidisciplinary team consisting pulmonologists, nurses, psychologists, physiotherapists and dietitians. After the initial evaluations, an eight-week, two-half-day outpatient, directly supervised PR program was implemented in line with personal needs. Also, individualized home exercise program was prescribed to all patients, three days a week. The PR program consists of exercise training, nutrition and psychosocial counseling, and, when necessary, supportive treatment and education of the patient and his family. The exercise training program consisted of endurance training and resistance training for the upper and lower extremities, based on recent guidelines [1,7]. Endurance training included 30 min of endurance exercise (15 min on a treadmill and 15 min on a stationary bicycle) at 85% of each patient’s VO2 peak calculated from the incremental shuttle walk test (ISWT) [7]. A 15-min warm-up and cool-down period was also included. Quadriceps resistance training entailed leg extensions performed using free weights two days per week for eight weeks according to 1-repetition maximum (1RM), starting at 45%–50% of 1RM for two sets, and 10 repetitions per set during the first 3–5 sessions, followed by 70% for three sets, and 10 repetitions per set during other sessions. Resistance training of the shoulder girdle and elbow muscles started at half a kilogram and increased to 1–1.5 kg with one set at 10 repetitions per set. During the PR sessions, the patients’ heart rate, blood pressure values and peripheral oxygen saturations were followed by physiotherapists.

#### 2.1.2 Structured follow-up program

Following the completion of the PR program, all patients were given a personalized home exercise program. The patients were explained and provided written and visual exercise education materials created in our center. Then the patients were invited for follow-up visits at three, six, and twelve months in the first year, and every six months in the second year. Home programs were modified to meet the needs of the patients whose PR assessments were performed at each control.

### 2.2 Outcome measures

Before and after the eight-week PR program, as well as at the 12th and 24th month controls, pulmonary function test, dyspnea sensation, exercise capacity, respiratory muscle srength, peripheral muscle strength, quality of life, body composition, and psychological status were recorded. Spirometry was performed to determine forced vital capacity (FVC), forced expiratory volume in one s (FEV1), and FEV1/FVC using a spirometer (AS-507, Minato Medical Science, Tokyo, Japan), in accordance with the American Thoracic Society-European Respiratory Society (ATS-ERS) guidelines [8]. Respiratory muscle strength was evaluated by measuring the maximal inspiratory pressure (MIP) using a Micro-RPM respiratory pressure meter (Care Fusion, Hoech-berg, Germany). MIP was measured in accordance with the recommendations of the ATS-ERS [9]. Test was repeated minimum of three times, and the best value was recorded. Exercise capacity was evaluated using the ISWT. The tests were performed according to field walking tests guidelines [10]. A handgrip test was performed using a hand dynamometer to assess the patients’ peripheral muscle strength. Also, 1-repetition maximum (1-RM) test is performed to evaluate the peripheral muscle strength. Health-related quality of life was assessed using the St. George’s Respiratory Questionnaire (SGRQ) [11] and dyspnea was assessed using the Medical Research Council (MRC) scale [12]. Hospital Anxiety and Depression (HAD) scores were used to assess psychological status [13].

MRC, ISWT, MIP, HG, SGRQ, HAD score results were used to evaluate long-term efficacy.

### 2.3 Statistical analysis

Statistical analyses were performed using the Statistical Package for the Social Sciences version 18.0 (SSPS, Chicago, IL, USA) software package. Normally-distributed numeric variables were expressed as mean and standard variation while nonnormally distributed variables were expressed as a median. Categorical variables were expressed as numbers and percentages (%). To determine if the variables were normally distributed, visual (histograms, probability plots) and analytical methods (Shapiro-Wilk, Skewness, and Kurtosis test) were used. The significance between the measurements of the variables at different time intervals was evaluated with the paired t-test. Baseline values and gains between the three groups were analyzed with a one-way ANOVA. Tukey HSD test and Games-Howell were used for post hoc analyses. The baseline PR levels and the values at the completion of the 8-week period were compared (p^post^). In addition, post-PR values and the data at the12th and 24th months were compared (p^12^, p^24^). For these, a paired t-test was used. A p-value of 0.05 was used to determine statistical significance.

## 3. Results

Of the 269 patients included in the study, 170 had COPD, 62 had bronchiectasis, and 37 had asthma. 195 patients were male and 74 were female. While 82.2% of patients with COPD were men, 62.2% of patients with asthma were women. The number of men and women in the bronchiectasis group was equal. While the mean age of all patients was 56, the COPD group had a mean age of 60, while the bronchiectasis and asthma groups had mean ages of 46 and 50, respectively (p < 0.001). The mean pack-years of smoking were higher in the COPD group than in the other two groups (p < 0.01). Percentages of active smoking were found to be 8.2%/6.5%/5.4% in COPD, bronchiectasis and asthma groups, respectively. Demographic data and baseline values of the patients are shown in Table 1. In an analysis of variance, the asthma group’s baseline MIP values were found to be statistically significantly higher (p = 0.020). The mean baseline HG value of COPD group was significantly higher than the other two groups (p = 0.010). Patients with asthma had a higher BMI than those with COPD and bronchiectasis (p < 0.001). All three groups had similar baseline quality of life, psychological state, dyspnea perception, and exercise capacity as determined by ISWT. Except for spirometry, the evaluation parameters after PR showed statistically significant improvement in all three groups (Tables 2–4).

The gains in the perception of dyspnea evaluated by MRC decreased from the first year compared to the levels after PR. Gains were maintained longer in the asthma group. However, MRC scores in all groups were lower than baseline levels even at the end of the second year. At the end of the first and second years, ISWT gains were preserved in the bronchiectasis and asthma groups. Although the exercise capacity in the COPD group decreased over time, it was approximately 30 m higher than the baseline value at the end of the second year. It was determined that MIP gains were maintained or even increased in all groups. While HG gains were maintained for 24 months in the COPD and bronchiectasis groups, they regressed to baseline levels at the end of 24 months in the asthma group. SGRQ gains decreased in all groups from the first year but were still better at the end of the second year than at baseline. The gains in anxiety and depression scores were mostly lost by the second year in all groups.

The difference between the measurement results of MRC, ISWT, MIP, HG, BMI, FFMI, SGRQ, HADa, and HADd parameters at 12 and 24 months and their baseline values were reported as PR gain. No difference was found comparing 12- and 24-month PR gains between the three groups using a one-way analysis of variance (Figure 2).

## 4. Discussion

In our study, we investigated how PR gains be maintained for 2 years in COPD, bronchiectasis, and asthma patients who completed a comprehensive outpatient multidisciplinary PR program and continued with structured follow-up programs. Except for anxiety and depression, PR gains were found to be sustained for two years, and patients with bronchiectasis and asthma had PR gains similar to COPD patients.

Previous studies have shown that gains in dyspnea perception can be maintained for 6–24 months in COPD patients after PR programs [14–16]. In a study of people with obstructive pulmonary disease (35 with asthma and 26 with COPD), it was observed that the gains in dyspnea perception evaluated with the Borg scale and the visual analogue scale (VAS) after the PR program were preserved, although partially decreased in the first year. The changes at the end of the first year were similar between the two groups [6]. In our study, the perception of dyspnea assessed by MRC was similar in all groups at the onset of PR (ANOVA p = 0.283). In the COPD and bronchiectasis groups, although the gains achieved after the first year decreased, they did not decrease to the baseline level, and in the asthma group, the gains in the perception of dyspnea were maintained at the end of the second year. In addition to the reversible nature of airway obstruction in asthma, it is thought that drug education given to patients within the scope of education programs increases treatment compliance and may contribute to asthma control.

According to a recent study, after a multidisciplinary, comprehensive, directly supervised outpatient PR program in COPD patients, dyspnea perception, exercise capacity, and quality of life improved, anxiety and depression levels decreased, the number of hospitalizations and i-BODE index decreased, and the gains were maintained for one year. In this study, it was concluded that the structured follow-up program applied at three-month intervals in the first year and then at six-month intervals was effective in maintaining the gains [17]. A meta-analysis showed that supervised exercise programs after primary PR were superior to usual care in maintaining gains in exercise capacity at 6 months but not at 12 months [18]. It has been concluded that the gains in exercise capacity continue to increase during the 96-week exercise training program consisting of directly supervised aerobic, upper extremity exercises, and inspiratory muscle training in COPD and that long-term programs can improve cardiovascular variables [19]. In a multicenter randomized controlled study, six-month evaluation results showed that asthmatic patients responded to PR in a similar way to COPD patients, but patients with asthma had a greater increase in walking distance [20]. In our study, exercise capacity gains were maintained for 24 months in patients with bronchiectasis and asthma. Although the ISWT averages of COPD patients did not regress to the baseline level after 24 months, it was revealed that their gains decreased statistically significantly from the first year. This result might be attributed to COPD patients’ older mean age as compared to the other two groups.

In a long-term trial in which inspiratory muscle exercises were combined with aerobic and upper extremity exercises in COPD patients, MIP values revealed a statistically significant increase at the end of 18 months [21]. In a study conducted without prescribing maintenance exercise, it was reported that the increase in inspiratory muscle strength was maintained for one year in asthma patients, while it was lost at the end of the first year in COPD patients [6]. In a research investigating the effectiveness of maintenance inspiratory muscle training in COPD, inspiratory muscle training was administered to a group for 30 min, three times a week, at 60% of monthly remeasured PImax values for 12 months in individuals with severe COPD. While the gains in dyspnea perception and exercise capacity were maintained for 1 year in this group, it was observed that there was a decrease in MIP values from the 9th month in the group that did not receive maintenance IMT [22]. In our study, MIP gains were increased in all groups after PR. In the COPD and asthma groups, gains in inspiratory muscle strength were preserved after 24 months, and in the bronchiectasis group, the mean MIP at 12 and 24 months increased statistically significantly compared to the PR end values. Following PR, our patients were given structured home exercise programs that were designed to their specific needs. Furthermore, comprehensive follow-up programs were established at the third and sixth months after PR, and then every six months thereafter, and our patients were evaluated during these controls, and maintenance programs were designed to meet their needs for two years. We believe that teaching inspiratory and expiratory respiratory muscle exercises, as well as bronchial hygiene procedures, and integrating them into maintenance programs are the most essential causes for the increase in MIP in bronchiectasis patients.

In our study, it was observed that the upper extremity muscle strength gains of the patients were maintained for 2 years in the COPD and bronchiectasis groups but decreased at the end of the 24th month in the asthma group. In Turkish society, it was determined that the upper extremity muscle strength was 33.4 kg in men and 19.5 kg in women at 50 years of age and 50% percentile [23]. In our study, the initial HG strength of patients with asthma was found to be 34.29 kg in men and 22.24 kg in women, and it was within normal limits. Although their FFMIs were within the normal range in both men and women, the BMI of the asthma group was significantly higher than the other two groups. Within the scope of our PR program, although there was a statistically significant decrease in BMI in asthmatic patients with dietetic counseling, it increased to baseline levels at the end of the 24th month. The failure to maintain the increase in HG levels may be related to the patient’s noncompliance with dietary recommendations and exercise programs during the maintenance period. Also, asthmatic patients may have used more systemic corticosteroids.

Previous studies have found contradictory results about the long-term benefits of PR on both quality of life and anxiety and depression in patients with obstructive pulmonary disease. Some researches found that improvements in quality of life and HAD might be sustained for 12–18 months following PR in COPD [24, 25]. Another study indicated that after 12 months, improvements returned to the baseline level [26]. A few studies have looked at the long-term effects of PR in non-COPD obstructive lung diseases. In a study, home-based PR was found to be associated with better long-term quality of life, but not with anxiety or depression in severe asthmatic patients [5]. In a meta-analysis, it was reported that no long-term effect on quality of life was found [27]. The short-term efficacy of PR on anxiety and depression in COPD has been demonstrated at levels of evidence I, II (strong recommendation) [28]. However, its long-term effectiveness is still controversial. In our study, the change in quality of life evaluated with SGRQ and anxiety-depression scores during 24 months after PR was the same in all three groups. SGRQ and HAD scores in all groups improved statistically at the end of PR. While the SGRQ scores in all three groups increased similarly at the 12th and 24th months, they were still statistically substantially below the baseline level at the 24th month (in all groups by t-test: p < 0.001). In addition, for COPD, the SGRQ score change between baseline and 24 months was above the minimal clinically important difference. Although HAD scores showed significant improvement at the end of PR, the decrease in anxiety and depression scores at 12 months was significantly lost and reached baseline values at 24 months. Quality of life in COPD patients has been correlated with a variety of factors, including physical capacity, psychological status, dyspnea, a lack of family and social support, anemia, and the ability to participate in daily activities [29, 30]. As in the research of Yohannes et al. [29], the deterioration in quality of life in our study might be explained by the negative influence of depression on quality of life.

We recommend that patients with the obstructive pulmonary disease be followed up in the third and sixth months and with six-month intervals thereafter as part of a structured follow-up program after PR.

Our study was a retrospective and single-center study. Medication control was not performed. Our study’s strengths are that it is a comprehensive, directly supervised, multidisciplinary program that involves long-term patient control with structured follow-ups. The most important limitation of our study was our knowledge of patients’ compliance with the maintenance program was mainly based on the patient’s statement.

## 5. Conclusion

Our study has shown that the efficacy of PR lasts up to 24 months in patients with COPD, bronchiectasis, and asthma. Only the anxiety and depression gains were preserved in the short term. The long-term efficacy of PR in patients with bronchiectasis and asthma was shown to be similar to that of COPD patients. As non-COPD obstructive lung disease, patients with bronchiectasis and asthma should be integrated into comprehensive multidisciplinary programs. We recommend structured follow-upto preserve the gains and determine the need for rerehabilitation.

## Figures and Tables

**Figure 2a:**
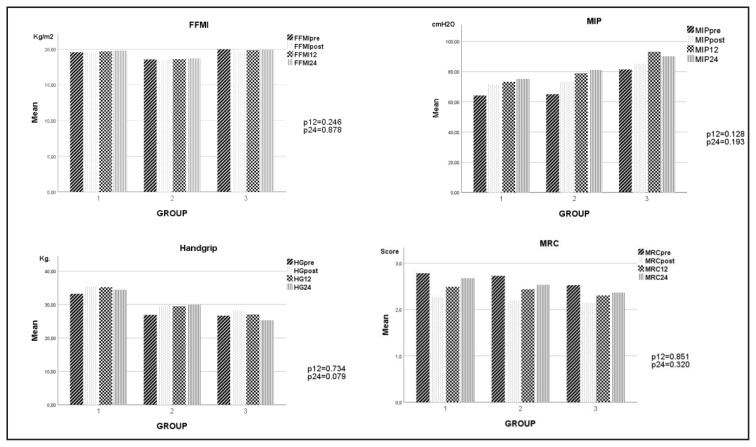
PR parameters at prePR, postPR, 12th and 24th months by groups. **1**: COPD, **2**: Bronchiectasis, **3**: Asthma, **p**: ANOVA significant value of PR gains at 12 and 24 months.

**Figure 2b:**
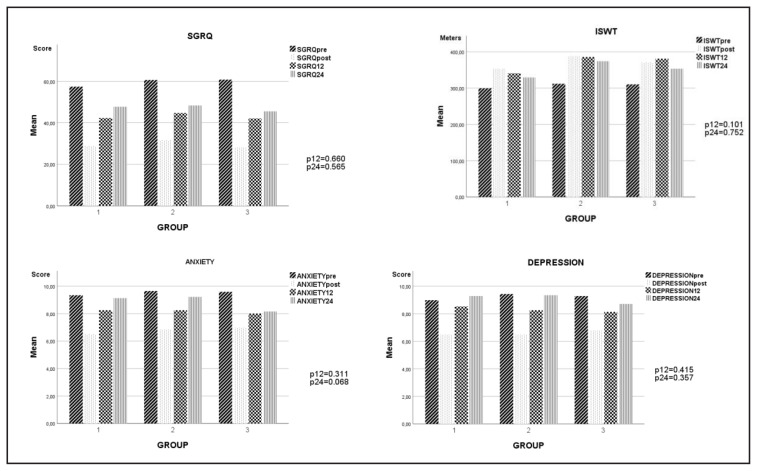
PR parameters at prePR, postPR, 12th and 24th months by groups. **1**: COPD, **2**: Bronchiectasis, **3**: Asthma, **p**: ANOVA significant value of PR gains at 12 and 24 months.

**Table 1:** Demographic and initial data.

	All patients (Mean ± SD)	COPD (Mean ± SD)	Bronchiectasis (Mean ± SD)	Asthma (Mean ± SD)	p	Post hoc tests		
						p^1^–^2^	p^1^–^3^	p^2^–^3^
**Sex m/f n (%)**	195/74 (72.5/27.5)	150/20 (88.2/11.8)	31/31 (50/50)	14/23 (37.8/62.2)	**<0.001**	**0.000** +	**0.000** +	0.469+
**Age (years)**	56.1 ± 12.1	60.7 ± 8.2	46.5 ± 14.8	50.8 ± 10.8	**<0.001**	**0.000** +	**0.000** +	0.224+
**Smoking History (p/year)**	41.8 ± 27.6	48.6 ± 25.5	10.7 ± 9.2	8.8 ± 5.2	**<0.001**	**0.000** +	**0.000** +	0.982+
**FVC (pred%)**	66.5 ± 19.6	64.9 ± 18.9	64.7 ± 19.9	76.8 ± 19.8	**0.003**	0.997*	**0.003** *	**0.009** *
**FEV1 (pred%)**	51.3 ± 21.2	47.9 ± 19.9	51.1 ± 20.2	67.4 ± 22.1	**<0.001**	0.563*	**0.000** *	**0.001** *
**FEV1/FVC**	61.2 ± 13.6	57.4 ± 13.4	65.1 ± 11.6	72.1 ± 9.6	**<0.001**	**0.000** *	**0.000** *	**0.023** *
**MRC**	2.7 ± 0.8	2.7 ± 0.8	2.7 ± 0.8	2.5 ± 0.7	0.283	1.000*	0.266*	0.371*
**ISWT (m)**	307.7 ± 128	301.9 ± 128.7	318.4 ± 130.4	316.6 ± 123.1	0.293	0.270*	0.815*	0.835*
**MIP (cmH2O)**	69.2 ± 20.5	66.7 ± 20.5	67.2 ± 17.9	80.8 ± 20.9	**0.020**	0.882*	**0.027** *	**0.028** *
**HG test (R) kg**.	30.4 ± 8.5	32.2 ± 7.9	27.0 ± 9.0	27.3 ± 8.2	**0.010**	**0.011** *	0.251*	0.827*
**HG test (L) kg**.	29.2 ± 8.4	31.1 ± 7.6	25.7 ± 8.5	25.5 ± 9.2	**0.002**	**0.003** *	0.273*	0.941*
**BMI (kg/m** **2** **)**	26.8 ± 6.2	26.3 ± 5.5	25.3 ± 6.9	31.6 ± 5.6	**<0.001**	0.503+	**0.000** +	**0.000** +
**FFMI (kg/m** **2** **)**	19.2 ± 2.5	19.4 ± 2.4	18.2 ± 2.7	19.9 ± 2.2	**0.001**	**0.003** *	0.485*	**0.002** *
**SGRQtotal**	58.7 ± 17.8	58.1 ± 17.7	59.2 ± 17.5	59.9 ± 19.2	0.837	0.930*	0.851*	0.976*
**HADa (Anxiety)**	9.4 ± 2.1	9.3 ± 2.3	9.7 ± 1.4	9.7 ± 2.0	0.271	0.406*	0.427*	0.984*
**HADd (Depression)**	9.1 ± 2.3	8.9 ± 2.3	9.3 ± 2.2	9.3 ± 2.5	0.471	0.686*	0.535*	0.938*

**BMI:** Body mass index, **FFMI:** Fat-free mass index, **HAD:** Hospital Anxiety and Depression, **HG:** Handgrip, **ISWT:** Incremental shuttle walk test, **MIP:** Maximal inspiratory pressure, **MRC:** Medical Research Council, **SGRQ:** St. George’s Respiratory Questionnaire, **p:** p value of ANOVA or Kruskal-Wallis test.

* Tukey HSD,

+ **:** Games-Howell (comparison between the groups),

1 COPD,

2 Bronchiectasis,

3 Asthma

**Table 2:** Follow-up parameters and p value of the COPD group according to initial data.

COPD	PostPR		12^th^ month		24^th^ month	
	Mean ± SD	p^post^	Mean ± SD	p^12^	Mean ± SD	p^24^
**FVC (pred%)**	66.6 ± 19.0	0.054	61.1 ± 19.5	**<0.001**	59.7 ± 20.2	**<0.001**
**FEV1 (pred%)**	49.3 ± 20.9	0.075	47.8 ± 20.8	**0.027**	46.4 ± 20.9	**<0.001**
**FEV1/FVC**	58.1 ± 14.3	0.270	60.4 ± 13.4	**0.003**	59.1 ± 13.3	0.299
**MRC**	2.2 ± 0.7	**<0.001**	2.5 ± 0.6	**<0.001**	2.6 ± 0.7	**<0.001**
**ISWT (m)**	355.1 ± 123.3	**<0.001**	341.7 ± 133.9	**0.014**	330.6 ± 147.8	**<0.001**
**MIP (cmH2O)**	73.5 ± 19.7	**<0.001**	74.1 ± 19.9	0.280	75.8 ± 20.0	0.102
**Handgrip (R) kg**.	34.6 ± 8.0	**<0.001**	34.8 ± 7.7	0.628	34.3 ± 8.4	0.146
**Handgrip (L) kg**.	33.4 ± 7.9	**<0.001**	33.6 ± 7.8	0.600	33.3 ± 8.1	0.286
**BMI (kg/m** **^2^** **)**	26.5 ± 5.4	**<0.001**	26.7 ± 5.5	**0.008**	26.7 ± 5.4	**0.033**
**FFMI (kg/m** **^2^** **)**	19.4 ± 2.3	0.956	19.7 ± 2.3	**0.019**	19.7 ± 2.5	**0.002**
**SGRQ**	29.6 ± 9.2	**<0.001**	42.4 ± 15.8	**<0.001**	48.5 ± 18.1	**<0.001**
**HADa (Anxiety)**	6.5 ± 2.3	**<0.001**	8.3 ± 1.7	**<0.001**	9.1 ± 2.0	**<0.001**
**HADd (Depression)**	6.5 ± 2.2	**<0.001**	8.5 ± 1.9	**<0.001**	9.2 ± 2.4	**<0.001**

**BMI:** Body mass index, **FFMI:** Fat-free mass index, **HAD:** Hospital Anxiety and Depression, **ISWT:** Incremental shuttle walk test, **MIP:** Maximal inspiratory pressure, **MRC:** Medical Research Council, **SGRQ:** St. George’s Respiratory Questionnaire, **p^post^:** p-value obtained by comparing the results before and after PR with the paired t-test, **p^12^:** p-value obtained by comparing the results at the end of PR and 12 months after PR with the paired t-test, **p^24^:** p-value obtained by comparing the results at the end of PR and 24 months after PR with the paired t-test.

**Table 3:** Follow-up parameters and p value of the bronchiectasis group according to initial data.

Bronchiectasis	postPR		12^th^ month		24^th^ month	
	Mean ± SD	p^post^	Mean ± SD	p^12^	Mean ± SD	p^24^
**FVC (pred%)**	66.1 ± 18.9	0.215	64.6 ± 20.5	0.218	62.7 ± 17.4	**0.017**
**FEV1 (pred%)**	53.2 ± 19.9	0.125	51.8 ± 20.2	0.108	50.9 ± 20.7	0.079
**FEV1/FVC**	66.3 ± 12.3	0.192	68.1 ± 12.9	0.491	68.5 ± 13.4	0.429
**MRC**	2.3 ± 0.6	**<0.001**	2.5 ± 0.7	**0.001**	2.5 ± 0.8	**0.001**
**ISWT (m)**	398.8 ± 121.8	**<0.001**	384.8 ± 125.3	0.259	390.9 ± 135.3	0.865
**MIP (cmH2O)**	77.1 ± 17.5	**<0.001**	81.6 ± 20.9	**0.007**	85.1 ± 18.9	**0.005**
**Handgrip (R) kg**.	29.4 ± 8.6	**<0.001**	30.2 ± 7.6	0.741	30.9 ± 9.7	0.614
**Handgrip (L) kg**.	29.3 ± 9.2	**<0.001**	28.8 ± 7.7	0.340	31.0 ± 10.5	0.428
**BMI (kg/m** **^2^** **)**	25.5 ± 6.6	**<0.001**	25.5 ± 6.3	0.731	25.8 ± 6.2	0.215
**FFMI (kg/m** **^2^** **)**	18.3 ± 2.9	0.577	18.2 ± 2.4	0.616	18.7 ± 2.6	**0.002**
**SGRQ**	30.8 ± 10.1	**<0.001**	42.7 ± 18.7	**<0.001**	47.7 ± 14.7	**<0.001**
**HADa (Anxiety)**	7.0 ± 1.9	**<0.001**	8.4 ± 1.9	**<0.001**	9.1 ± 1.8	**<0.001**
**HADd (Depression)**	6.7 ± 1.8	**0.001**	8.6 ± 2.5	**<0.001**	9.2 ± 2.0	**<0.001**

**BMI:** Body mass index, **FFMI:** Fat-free mass index, **HAD:** Hospital Anxiety and Depression, **ISWT:** Incremental shuttle walk test, **MIP:** Maximal inspiratory pressure, **MRC:** Medical Research Council, **SGRQ:** St. George’s Respiratory Questionnaire, **p^post^:** p-value obtained by comparing the results before and after PR with the paired t-test, **p^12^:** p-value obtained by comparing the results at the end of PR and 12 months after PR with the paired t-test, **p^24^:** p-value obtained by comparing the results at the end of PR and 24 months after PR with the paired t-test.

**Table 4:** Follow-up parameters and p value of the asthma group according to initial data.

Asthma	postPR		12^th^ month		24^th^ month	
	Mean ± SD	p^post^	Mean ± SD	p^12^	Mean ± SD	p^24^
**FVC (pred%)**	77.1 ± 18.6	0.955	77.1 ± 18.6	0.542	73.1 ± 19.3	0.086
**FEV1 (pred%)**	68.3 ± 22.4	0.829	67.7 ± 19.7	0.251	66.2 ± 21.9	0.361
**FEV1/FVC**	72.6 ± 10.4	0.695	72.2 ± 10.1	0.395	73.3 ± 11.1	0.414
**MRC**	2.1 ± 0.5	**0.009**	2.3 ± 0.5	0.083	2.3 ± 0.6	0.158
**ISWT (m)**	379.4 ± 122.5	**<0.001**	380.6 ± 133.3	0.770	360.4 ± 127.6	0.311
**MIP (cmH2O)**	86.9 ± 27.2	**0.022**	95.8 ± 25.8	0.111	90.8 ± 23.1	0.294
**Handgrip (R) kg**.	29.4 ± 8.3	**<0.001**	29.5 ± 9.2	0.365	26.5 ± 7.2	**0.050**
**Handgrip (L) kg**.	28.1 ± 7.8	**<0.001**	28.3 ± 8.9	0.358	25.5 ± 7.7	**0.037**
**BMI (kg/m** **^2^** **)**	31.2 ± 5.2	**<0.001**	31.4 ± 5.4	0.596	31.6 ± 5.5	0.074
**FFMI (kg/m** **^2^** **)**	19.7 ± 2.1	**0.013**	19.8 ± 2.1	0.338	19.9 ± 2.2	0.150
**SGRQ**	28.3 ± 8.6	**<0.001**	42.1 ± 18.3	**<0.001**	45.50 ± 17.3	**<0.001**
**HADa (Anxiety)**	7.0 ± 1.7	**<0.001**	8.0 ± 1.6	**0.025**	8.9 ± 1.9	**0.011**
**HADd (Depression)**	6.9 ± 1.8	**<0.001**	8.1 ± 2.2	**0.018**	8.7 ± 2.1	**0.001**

**BMI:** Body mass index, **FFMI:** Fat-free mass index, **HAD:** Hospital Anxiety and Depression, **ISWT:** Incremental shuttle walk test, **MIP:** Maximal inspiratory pressure, **MRC:** Medical Research Council, **SGRQ:** St. George’s Respiratory Questionnaire, **p^post^:** p-value obtained by comparing the results before and after PR with the paired t-test, **p^12^:** p-value obtained by comparing the results at the end of PR and 12 months after PR with the paired t-test, **p^24^:** p-value obtained by comparing the results at the end of PR and 24 months after PR with the paired t-test.
